# Functional changes between seasons in the male songbird auditory forebrain

**DOI:** 10.3389/fnbeh.2013.00196

**Published:** 2013-12-19

**Authors:** Geert De Groof, Colline Poirier, Isabelle George, Martine Hausberger, Annemie Van der Linden

**Affiliations:** ^1^Department of Biomedical Sciences, Bio-Imaging Lab, University of AntwerpAntwerp, Belgium; ^2^UMR6552 - Ethologie Animale et Humaine, Université Rennes 1 - CNRSRennes, France

**Keywords:** European starling, caudomedial nidopallium, NCM, seasonal plasticity, auditory perception, functional magnetic resonance imaging, lateralization, songbird

## Abstract

Songbirds are an excellent model for investigating the perception of learned complex acoustic communication signals. Male European starlings (*Sturnus vulgaris*) sing throughout the year distinct types of song that bear either social or individual information. Although the relative importance of social and individual information changes seasonally, evidence of functional seasonal changes in neural response to these songs remains elusive. We thus decided to use *in vivo* functional magnetic resonance imaging (fMRI) to examine auditory responses of male starlings that were exposed to songs that convey different levels of information (species-specific and group identity or individual identity), both during (when mate recognition is particularly important) and outside the breeding season (when group recognition is particularly important). We report three main findings: (1) the auditory area caudomedial nidopallium (NCM), an auditory region that is analogous to the mammalian auditory cortex, is clearly involved in the processing/categorization of conspecific songs; (2) season-related change in differential song processing is limited to a caudal part of NCM; in the more rostral parts, songs bearing individual information induce higher BOLD responses than songs bearing species and group information, regardless of the season; (3) the differentiation between songs bearing species and group information and songs bearing individual information seems to be biased toward the right hemisphere. This study provides evidence that auditory processing of behaviorally-relevant (conspecific) communication signals changes seasonally, even when the spectro-temporal properties of these signals do not change.

## Introduction

Birdsong, like human speech, is a learned vocal behavior whose function is to communicate with others. It is also a signal that has a sender and a receiver and whose meaning and function is asserted by the effect on the receiver and not only by the signal's structure (see review by Scott-Phillips, 2008). The same song may indeed convey different meanings according to the context or the receiver. In cowbirds (*Molothrus ater ater*) for example, the same song types may elicit either aggression in males or attraction in females (West et al., 1997). In the European starling (*Sturnus vulgaris*), the high-pitched trills that characterize male song sequences during the breeding season have been observed to be involved in female attraction during the breeding season but not during the non-breeding season (Verheyen, 1970; Adret-Hausberger and Jenkins, 1988; Henry et al., 1994). Interestingly, the neural response to these trills in female starlings' primary auditory area is higher during the breeding season than during the non-breeding season (Cousillas et al., 2013).

Auditory responses are seasonally regulated in a number of species and at different levels of the auditory pathway (reviewed by Maney and Pinaud, 2011). Although most studies focused on females, for whom the behavioral relevance of male song changes dramatically according to season (Maney et al., 2006; Sanford et al., 2010), some studies in males have shown a sound-induced ZENK response in the auditory forebrain that was selective for conspecific over control sounds (Matragrano et al., 2013) or hetero-specific songs (Phillmore et al., 2011) only when the males were in breeding condition. As far as we know, no one has ever looked at seasonal changes in selectivity of neural responses *within* conspecific song, by testing responses to different song types of the same species. Although canaries (*Serinus canaria*) have been studied for seasonal changes in auditory responses to songs (Alliende et al., 2012), their song (acoustic) structure varies dramatically with the season. It is therefore difficult to conclude if the changes in auditory responses seen in canaries are due to changes in song behavioral relevance or to changes in acoustic feature perception. Thus, we decided to study a songbird species that would allow us to separate these factors: the European starling.

The European starling is a highly social, seasonally breeding bird and one of only a few temperate climate songbird species that display a high song rate throughout most of the year, including during the non-breeding season (Eens, 1997) when plasma T levels are basal (Riters et al., 2000; Van Hout et al., 2009). This means that starlings sing in a variety of social contexts going from large groups of starlings feeding in flocks of 10–500 individuals and sleeping in night roosts of up to 3 million individuals to small groups breeding in colonies of 3–15 nests (Verheyen, 1970; Hausberger, 1997). The time spent in each of these groups varies seasonally, with starlings spending most of their time in large groups during the non-breeding season whereas they spend most of their time in pairs or in small groups during the breeding season. Such a variation in social organization and behavior is likely to imply a change in communication and in the role that song structures play in vocal/social recognition.

Starlings produce songs that correspond to distinct levels of discrimination. These songs can be divided into two main categories: loud, discrete whistles that are mainly used in vocal interactions at long distance, and a long, continuous warbling song that is produced mostly at low intensity and that includes varied mimicries of environmental sounds (Eens et al., 1989; Hausberger, 1991). Amongst whistles, one can distinguish whistles that are universally shared by all males and whose basic acoustic structure is similar in all populations studied (species-specific whistles) (Adret-Hausberger, 1982; Hausberger et al., 2000), and whistles that are characteristic of each starling in its colony (individual whistles) (Hausberger, 1997).

Species-specific whistles show local variations that give rise to a complex system of dialects (Adret-Hausberger, 1983), and they are involved in vocal interactions between males in a variety of contexts, including roosts and flocks (Adret-Hausberger, 1982). They may play a role in spacing when males settle in their colony (which may occur well-before breeding starts) (Henry et al., 1994), and they can be heard throughout the year in sedentary populations (Adret-Hausberger, 1984). Playback experiments using dialectal variants of these whistles have shown that male starlings discriminate their own variant from an unknown variant: they respond more often and more quickly to the familiar dialect (Adret-Hausberger, 1982). However, even if an unfamiliar variant is broadcast, males do respond by using their own variant of the whistle. This species-specific recognition seems to be based on key acoustic features whose modification suppresses vocal responses (Hausberger et al., 2000). In addition to species-specific whistles, field observations of hundreds of starlings across four continents have shown that, within a colony, each starling has a unique repertoire of individual whistle types (Adret-Hausberger et al., 1990). In captivity, these individual whistles can be shared by same-sex social partners that are closely associated (Hausberger et al., 1995). Although these whistles do not evoke vocal responses, playback experiments have shown that starlings are able to discriminate whistles of familiar individuals (Adret-Hausberger, 1982; Hausberger et al., 1997). Finally, warbling is a relatively soft, continuous song that is produced in long sequences made of highly individual motifs (also called variable motifs; motifs are fixed repeatable combinations of notes) at the beginning of a sequence and of some species-specific motifs (click motifs and high-pitched trills) that are common to all male starlings at the end of a sequence (Adret-Hausberger and Jenkins, 1988; Eens et al., 1989; Adret-Hausberger et al., 1990; Hausberger et al., 1995). Warbling is involved in short-distance communication, especially between males and females, and it is thought to play a role in mate choice (Eens et al., 1990, 1991). Warbling motifs have been shown to be the basic unit of individual recognition in starlings (Gentner and Hulse, 1998, 2000; Gentner, 2004), although warbling sequences also contain a few motifs that are common to all males (Adret-Hausberger and Jenkins, 1988; Eens et al., 1989, 1991).

Overall, starling song thus contains universal, species-specific song types that are mainly involved in remote social interactions between males and that convey general information, as well as song types that bear individual information and that are involved in close social interactions. The use of these two types of songs varies according to the breeding status, with for example a decrease in species-specific whistles when male starlings succeed in breeding (Henry et al., 1994). However, and although whistling activity and warbling sequences' organization show seasonal variations, no seasonal variation has been observed in the acoustic structure of either the whistles or the warbling motifs (Adret-Hausberger, 1984; Adret-Hausberger and Jenkins, 1988; Adret-Hausberger et al., 1990). Starlings' communication system therefore provides a unique opportunity to test whether brain processing of the same song changes with the season (and hence with the behavioral relevance of this song).

The most likely candidate region where such changes may occur is the telencephalic auditory region called the caudomedial nidopallium (NCM). We have shown that this region displays anatomical seasonal changes in male starling (De Groof et al., 2009). Also, the activation of NCM neurons is the greatest when birds are exposed to conspecific songs as compared to heterospecific songs or non-song stimuli (Mello et al., 1992; Chew et al., 1996; Stripling et al., 1997; Grace et al., 2003; Theunissen et al., 2004). In starlings, in addition to increasing from non-specific to species-specific sounds, neuronal activation increases from sounds bearing species-specific information to sounds bearing individual information (George et al., 2008). This increase in neuronal activation seems to rely more on the behavioral relevance of sounds than on their acoustic structure as failure to correctly use songs whose elementary acoustic structure is otherwise species-typical leads to undifferentiated neural responses in NCM (George et al., 2010). Indeed, starlings that could hear but not interact with adults during early life are unable to differentiate individual whistles and warbling motifs not only in their vocalizations but also in their neural (NCM) responses to these songs, independently of the structural differences between these two types of songs. Although they do produce individual whistles and warbling motifs whose acoustic morphology is species-typical, these songs are not produced in species-typical sequences and NCM responses to these songs do not differ like they normally differ in wild-caught starlings (George et al., 2008). NCM neurons therefore appear to encode the behavioral relevance of songs. We wondered if the processing of behaviorally relevant songs changes throughout the year in NCM.

To study this question, we investigated in the European starling the seasonal change in the processing of vocalizations that clearly convey different levels of information. Using functional magnetic resonance imaging (fMRI), we investigated the neural representation of these vocalizations during and outside the breeding season. Comparison of neural activity triggered by songs bearing species-specific and group information vs. songs bearing individual information revealed a differentiated response between these types of song in NCM. More importantly, this differentiated response showed a seasonal change in a sub-region of NCM, and it appeared to be lateralized.

## Materials and methods

### Ethics statement

Experimental procedures were in agreement with the Belgian laws on the protection and welfare of animals and were approved by the ethical committee of the University of Antwerp (License number: 2009-04).

### Subjects

Twelve wild-caught male European starlings (*Sturnus vulgaris*, ±75–95 g) were used in this experiment. Birds were caught as adults in Normandy (France) three and a half years (November 2006) before the experiments. They were first kept together in an indoor aviary with an artificial light-dark cycle simulating the natural photoperiod and later in outdoor aviaries at the University of Rennes 1 (Rennes, France) with food and water *ad libitum*. During the experiments at the University of Antwerp (Belgium), they were housed in two indoor cages (1.40 × 2.20 × 2.10 m) under an artificial light-dark cycle simulating the natural photoperiod. In each cage, males were put together with other wild-caught males and females (cage 1: 9 males and 6 females; cage 2: 8 males and 7 females). Food and water were available *ad libitum*. All birds were individually marked with color bands.

### Stimuli and stimulation device

**Figure 1:**
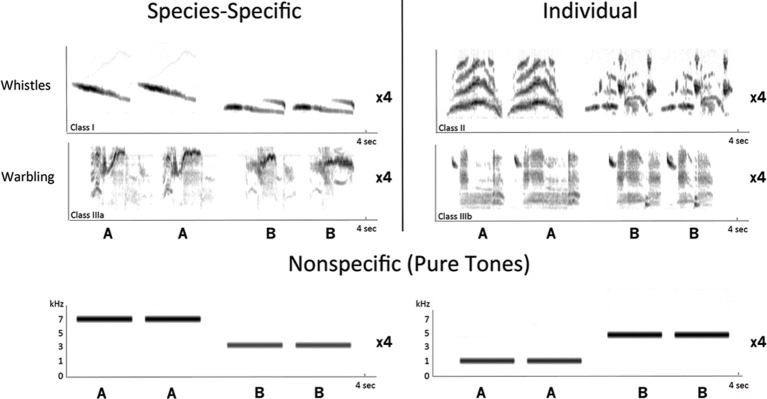
**Sonograms of the stimuli used in the experiment.** Stimuli were divided according to the level of social information that they convey: stimuli bearing species-specific and group information consisted of 2 species-specific whistles and 2 species-specific warbling motifs; stimuli bearing individual information consisted of 2 individual whistles and 2 individual warbling motifs. Artificial nonspecific stimuli consisted of pure tones (1, 3, 5, and 7 kHz). The depicted sequence of songs or sounds (AABB) was repeated four times per stimulus, bringing the total length of each stimulus to 16 s.

Both artificial non-specific sounds and natural starling sounds corresponding to the distinct types of starling songs described in the introduction (see also Hausberger, 1997) were used. In total, 5 types of stimuli were used (Figure 1):

– Artificial *pure tones* (PT) stimuli (one stimulus made out of pure tones at 7 and 3 kHz and another one made out of pure tones at 1 and 5 kHz, both stimuli interleaved with silence periods of 0.5 s).
– Four types of starling song stimuli consisting of songs bearing either species-specific and group information (songs shared by all males) or individual information, each recorded from 2 different starlings unknown to the birds (hence all song stimuli can be considered to be novel or unfamiliar):
  - *Species-specific whistles* [an inflection theme and a simple theme as described by (Hausberger, 1997)].
  - *Individual whistles* (Hausberger, 1997).
  - *Species-specific warbling* (high-pitched trills taken from the terminal, non-individual part of warbling that is found in the repertoire of all male starlings).
  - *Individual warbling* (individual motifs taken from the initial, individual part of warbling).

The total duration of each stimulus was 16 s and consisted of two elements (A and B) of the same type of song repeated in a [(AABB) × 4] fashion (Figure 1). The intensity of each song was normalized (in terms of matched root-mean-square) before being integrated into the complete stimulus (songs and silence periods). These manipulations were done using Praat software (University of Amsterdam, The Netherlands).

During imaging experiments, auditory signals were presented to the birds with magnetless dynamic speakers connected to an amplifier as described by Van Meir et al. (2005). Stimulus presentation was controlled by Presentation software 0.76 (Neurobehavioral Systems). Frequencies between 2500 and 5000 Hz are known to be enhanced in the setup (Poirier et al., 2009). To compensate for this artificial enhancement, an equalizer function was applied to each stimulus using WaveLab software (Steinberg). The function consisted of a Gaussian kernel with the following parameters: maximum amplitude: −20 dB, centered on 3750 Hz, width: 0.05 octaves (corresponding to the range 2500–5000 Hz). During the experiments, stimuli were delivered to both ears with a sampling frequency of 22050 Hz and their global intensity was 67 dB sound pressure level. By comparison, the magnet noise was 65 dB.

### Experimental design

The experiments were conducted once during the breeding season (Spring: 29 March–15 April 2010) when birds are photostimulated, their gonads fully developed, and testosterone levels high and were repeated for the same birds during the non-breeding season (Fall: 27 September–9 October 2010) when birds have become photorefractory, their gonads fully regressed, and testosterone levels are low. The beak color of all subjects was assessed during both spring and fall. Beak color in European starlings is dependent on plasma T (Dawson and Howe, 1983; Ball and Wingfield, 1987). It changes from yellow in spring (when plasma T levels are higher) to black in fall (when plasma T levels are basal). It was recorded on an arbitrary scale of 0 (bill entirely black, from base to tip) to 5 (bill entirely yellow) (De Ridder et al., 2002). In spring, the beak of all males (*N* = 12) was yellow (4.0 ± 0.51; range: 3.5–4.8). During fall, the beak of all males (*N* = 12) was entirely black (score of zero in all cases; Related-samples Wilcoxon Signed Ranks test, *p* = 0.007).

Each fMRI experiment consisted of 2 sessions according to the level of information conveyed by the stimuli used: a session with songs conveying species-specific and group information and a session with songs conveying individual information. Each session included 3 stimuli: PT, whistles (either species-specific or individual whistles) and warbling (either species-specific or individual motifs) (Figure 1). The PT stimulus was the same in both sessions. Each session consisted of an ON/OFF block design alternating auditory stimulation periods (ON blocks) with resting periods (OFF blocks). Each block (ON and OFF) lasted 16 s, which corresponds to the acquisition time of 2 images. Each stimulus type was presented 42 times, resulting in the acquisition of 84 images per stimulus and per subject. The presentation and session order of the conditions were counterbalanced within and between subjects.

### Anesthesia and physiology monitoring

During the experiment, birds were anesthetized with an intramuscular injection in the chest of 0.4 ml of a mixture containing 10 ml of medetomidine (1 mg/ml, Domitor, Orion, Finland) and 0.5 ml of ketamine (50 mg/ml, Ketalar, Parke-Davis, Belgium). Body temperature was continuously monitored with a cloacal temperature probe and maintained at 41.5 ± 0.5°C by a feedback controlled warm air heating system (SA-Instruments). Respiration rate and amplitude were constantly monitored with a small pneumatic sensor (SA-Instruments) positioned under the bird.

### Image acquisition

Imaging was performed on a horizontal MR system (Pharmascan 70/16 US, Bruker Biospin, Germany) with a magnetic field strength of 7 Tesla. Specifications of the coils used for the experiment can be found in Van Meir et al. (2005). BOLD fMRI data were acquired using a T_2_-weighted Fast Spin Echo sequence [echo time/repetition time: 60/2000 ms] (Poirier et al., 2010). Each whole-brain volume contained 15 sagittal slices, 1 mm thick, with a gap of 0.066 mm between slices. In-plane resolution was 0.34 × 0.34 mm^2^ and matrix size was 64 × 64 voxels. Anatomical three-dimensional (3D) images required for localization of the functional data (see below) were obtained for each bird using a RARE T_2_-weighted sequence with TE/TR: 60/2000 ms. Voxel size was 0.085 × 0.085 × 0.085 mm^3^ and matrix size was 256 × 256 × 256 voxels.

### Image processing

Intra-individual head motion (from the 2 sessions) was corrected using a six-parameter rigid body spatial transformation using the Statistical Parametric Mapping toolbox (SPM8; Wellcome Department of Cognitive Neurology, London, UK; http://www.fil.ion.ucl.ac.uk/spm/). The realigned fMRI images for each subject were then coregistered to each individual anatomical 3D dataset. In parallel, the 3D dataset was spatially normalized using SPM8 with a high-resolution *ex-vivo* starling MRI image/atlas that we developed in our lab (unpublished data). The transformation matrix of this spatial normalization was then applied to the realigned and co-registered functional data, resulting in functional data precisely coregistered to the atlas dataset. Finally, functional data were smoothed with a 0.68 mm width Gaussian kernel.

### Statistical analysis

Statistical voxel-based analyses were performed using a mass-univariate approach based on the General Linear Model implemented in SPM8. Data were filtered with a high-pass filter of 352 s. Model parameters were then estimated using a classical restricted maximum likelihood algorithm.

**Figure 2:**
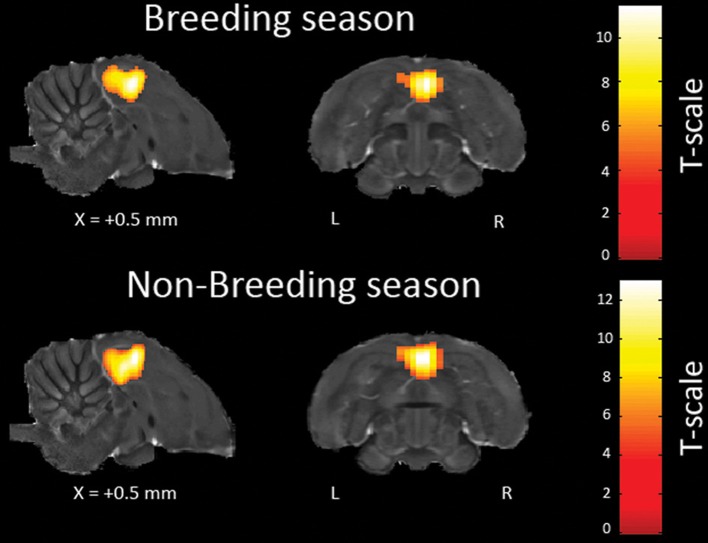
**Activations induced in the same individual by all the auditory stimuli (vs. rest) across seasons.** The statistical parametric maps (unilateral one sample *t*-test) are superimposed on anatomical sagittal and axial images coming from the high-resolution starling image. They illustrate the bilateral activation of Field L, the equivalent of the mammalian primary auditory cortex, and the (caudally and frontally) adjacent secondary auditory regions. The position of the slice along the X (left/right) axis is indicated (the + sign indicates that results are from the right hemisphere). *T*-values are color coded according to the scales displayed on the right side of the figure. Only voxels in which the *t*-test was found significant (*p*-value < 0.05, corrected for multiple comparisons at the whole brain level) are displayed.

The main effect of each stimulus (as compared to the rest period) was computed in each voxel, for each subject. In a second step, a group analysis was performed on the effects identified by the previous analysis. The individual analyses revealed a BOLD response triggered by the auditory stimuli in the bilateral Field L and NCM in 8 of the 12 birds for each season (Figure 2). This success rate is similar to the one obtained in previous spin-echo fMRI experiments on zebra finches (Poirier et al., 2010, 2009). The subsequent group analyses were performed only with these birds.

The statistical group analysis was restricted to some *a priori* defined regions of interest (ROI). Song control nuclei: HVC (used as a proper name; Reiner et al., 2004), the nucleus robustus of the arcopallium (RA), area X, and the lateral magnocellular nucleus of anterior nidopallium (LMAN); Auditory regions: the dorsal part of the lateral mesencephalic nucleus (MLd), nucleus Ovoidalis, Field L, the caudomedial mesopallium (CMM), NCM; and because it has been shown for responding to song in female zebra finches also the hippocampus (Bailey et al., 2002) (Mello and Clayton, 1994; however, see Stripling et al., 2001). NCM was delineated using Field L as rostral border, the cerebellum as caudal border and the lateral ventricle as ventral and dorsal borders. The boundaries of NCM in the lateral direction are unknown. The lateral border was set at 1.6 mm lateral to the midline. These lateral boundaries incorporate the NCM as defined in previous experiments on starlings (Gentner, 2004; Van Meir et al., 2005; George et al., 2008). CMM was defined as the region located dorso-rostral to Field L, ventral to the lateral ventricle and dorsal to the Lamina mesopallialis. Because they were too small to be sampled by at least one sagittal slice, dorsolateral nucleus of the medial thalamus and the nucleus interface of the nidopallium were not investigated.

We first identified in the predefined ROI, within each season, voxels that displayed a significant differential effect between SPEC and INDIV stimuli [(SPEC—PT) vs. (INDIV—PT)] in a 3 × 2 repeated measures ANOVA (1st within factor: three stimuli; 2nd within factor: SPEC and INDIV sessions). In other words, the response in these voxels to each of the two types of song stimuli (relative to the response of PT) was different for each session, or the difference in response between sessions was not the same for the two types of stimuli. The PT stimulus was used as a control for identifying differences between sessions. In a second step, we focused exclusively on those voxels that showed a significant session effect and we investigated the nature of this general effect by performing *post-hoc* paired *t*-tests per stimulus between sessions.

We then looked for seasonal changes by testing for a potential interaction between seasons and the differential effect between SPEC and INDIV stimuli [(INDIV—PT) vs. (SPEC—PT)]_breeding_ vs. [(INDIV—PT) vs. (SPEC—PT)]_non−breeding_. Although the same birds were measured repeatedly over the seasons, 3 birds did not show a successful fMRI in both seasons. Further analysis was therefore performed excluding these subjects. We first identified in the predefined ROI, voxels that displayed a significant “session × season” interaction in a 3 × 2 × 2 repeated measures ANOVA (1st within factor: 3 stimuli; 2nd within factor: 2 sessions; 3rd within factor: 2 seasons). In a second step, *post-hoc* paired *t*-tests were then performed only on those voxels that showed a significant interaction, to compare responses to stimuli obtained during each season. Because statistical tests were performed on a voxel basis, many tests were made; *p*-values were therefore adjusted to the number of independent tests performed. This was done using the Family Wise Error method. This method uses the Random Field Theory to calculate the number of independent tests. It takes into account the number of voxels but also the amount of auto-correlation among the data.

Finally, in order to compare responses obtained in both hemispheres, we calculated for each subject the differential effect between INDIV and SPEC stimuli [(INDIV—PT)–(SPEC—PT)] for both seasons. This was done in the two right NCM clusters found to be significantly differentially activated in the group analysis during the breeding season (see Results) and in their mirrored counterpart in the left hemisphere. These differential effects were then compared across hemispheres using two-tailed paired *t*-tests. Differences were considered as statistically significant when *p* < 0.05.

## Results

Every male starling underwent two (identical) fMRI experiments, one during the breeding season and one during the non-breeding season. Each fMRI experiment consisted of two sessions in which auditory responses in the brain were measured to the presentation of either songs conveying species-specific and group information (SPEC session) or individual information (INDIV session), and synthetic pure tones (PT, both sessions).

### Differential BOLD response between SPEC and INDIV stimuli during the breeding season

**Figure 3:**
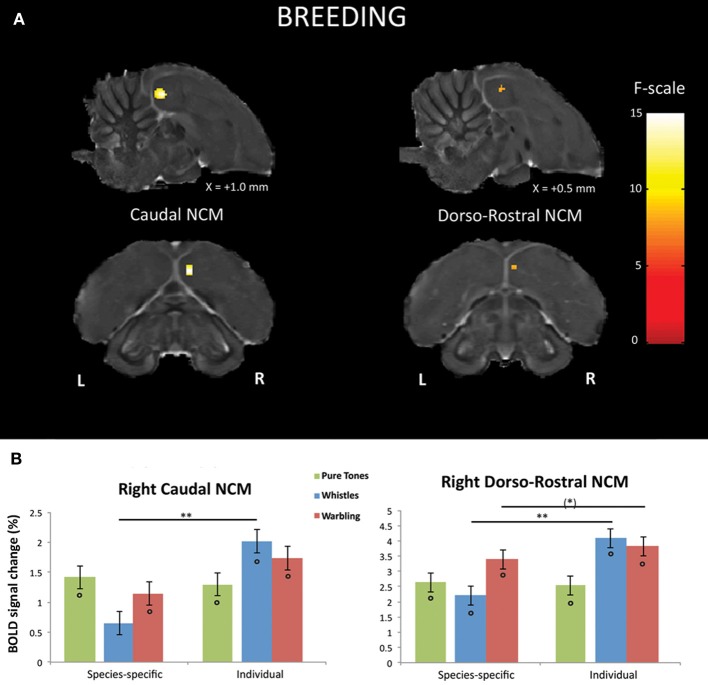
**Neural substrates for differential song processing during the breeding season. (A)** Superimposition of the statistical results to anatomical sagittal and axial images coming from the high-resolution starling image. The position of the slice along the X (left/right) axis is indicated (the + sign indicates that results are from the right hemisphere). Results are of those voxels displaying a significant differential response between songs conveying species-specific and group information (SPEC) and songs conveying individual information (INDIV). *F*-values are color coded according to the scale displayed on the right side of the panel. **(B)** Estimates of the relative (vs. rest) response amplitude (±SEM) of neural activations elicited by the different stimuli in the clusters illustrated in **(A)** (the values have been extracted from the voxel with the maximum *T*-value). The zero level corresponds to the mean activation level during rest periods (exposure to scanner noise). Circles indicate statistically significant differences between stimuli vs. rest. Stars indicate statistical significance of comparison between individual song stimuli and species-specific song stimuli (^*^p < 0.10; ^**^p < 0.001).

First, we looked, within the breeding season, for brain regions in which a differential response between SPEC and INDIV stimuli was observed. Only right NCM showed such differential responses. Two separate clusters of voxels could be identified in right NCM: a caudal NCM cluster (*F*_max_ i.e., voxel presenting the maximal *F*-value among all significant voxels of the cluster = 14.54, *p* = 0.024) and a dorso-rostral NCM cluster (*F*_max_ = 14.51, *p* = 0.025) (Figure 3A).

Further comparisons in right caudal NCM revealed a significantly greater neural activity induced by INDIV whistles compared to SPEC whistles (*t*_max_ i.e., voxel presenting the maximal *t*-value among all significant voxels of the cluster = 5.25; *p* < 0.001) (Figure 3B). INDIV warbling was not statistically different from SPEC warbling (*t*_max_ = 2.38; *p* = 0.122) and no significant difference between sessions was observed for PT (*t*_max_ = 0.47; *p* = 0.801) (Figure 3B). Analysis of the differential effect between SPEC and INDIV stimuli in dorso-rostral NCM showed that it resulted from a significantly greater neural activity induced by INDIV whistles compared to SPEC whistles (*t*_max_ = 5.10; *p* < 0.001). INDIV warbling compared to SPEC warbling showed a trend (*t*_max_ = 2.65; *p* = 0.071) (Figure 3B). No significant difference between sessions was observed for PT (*t*_max_ = 0.25; *p* = 0.820) (Figure 3B).

### Differential BOLD response between SPEC and INDIV stimuli during the non-breeding season

**Figure 4:**
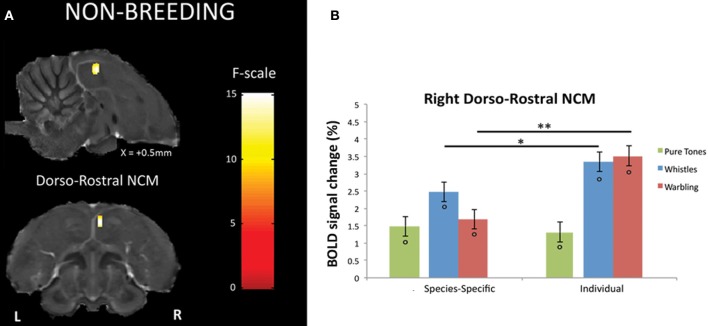
**Neural substrates for differential song processing during the non-breeding season. (A)** Superimposition of the statistical results to anatomical sagittal and axial images coming from the high-resolution starling image. The position of the slice along the X (left/right) axis is indicated (the + sign indicates that results are from the right hemisphere). Results are of those voxels displaying a significant differential response between songs conveying species-specific and group information (SPEC) and songs conveying individual information (INDIV). *F*-values are color coded according to the scale displayed on the right side of the panel. **(B)** Estimates of the relative (vs. rest) response amplitude (±−SEM) of neural activations elicited by the different song stimuli in the cluster illustrated in **(A)** (the values have been extracted from the voxel with the maximum *F*-value). The zero level corresponds to the mean activation level during rest periods (exposure to scanner noise). Circles indicate statistically significant differences between stimuli vs. rest. Stars indicate statistical significance of comparison between species-specific song stimuli and individual song stimuli (^*^p < 0.05; ^**^p < 0.001).

Within the non-breeding season (as within the breeding one), only right NCM showed a differential response between SPEC and INDIV stimuli. This time, however, only one cluster displayed such differential response: a dorso-rostral NCM cluster (*F*_max_ = 15.11, *p* = 0.029) (Figure 4A). Further comparisons in the right dorso-rostral NCM revealed a significantly greater neural activity induced by both INDIV whistles (*t*_max_ = 2.01; *p* = 0.011) and INDIV warbling (*t*_max_ = 4.36; *p* < 0.001) compared to SPEC whistles and SPEC warbling, respectively (Figure 4B). No significant difference between sessions was observed for PT (*t*_max_ = 0.37; *p* = 0.447) (Figure 4B).

### Seasonal effect

**Figure 5:**
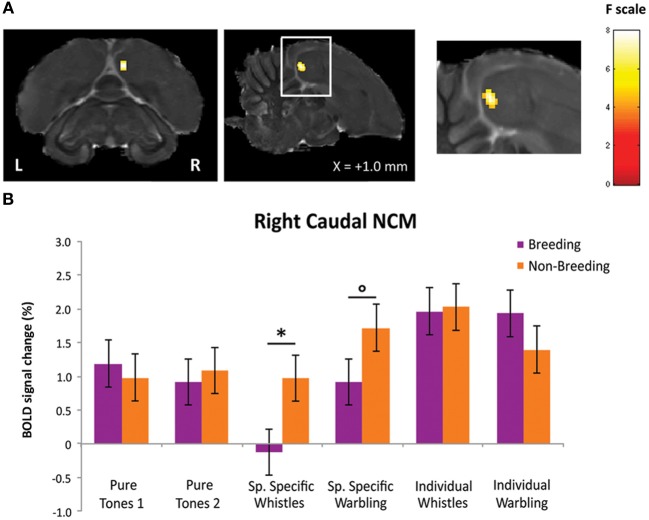
**Effect of season on differential song processing. (A)** Statistical map of voxels displaying a significant difference in differential song processing between breeding and non-breeding season [(INDIV—PT) vs. (SPECT—PT)]_breeding_ vs. [(INDIV—PT) vs. (SPECT—PT)]_non-breeding_ (*F*-test from a repeated measures ANOVA; *N* = 5). *F*-values are color coded according to the scale displayed on the right. The position of the slice along the X (left/right) axis is indicated (the + sign indicates that results are from the right hemisphere). **(B)** Estimates of the relative (vs. rest) response amplitude (+ SEM) of neural activations elicited by the different song stimuli in the cluster illustrated in **(A)** (values from the voxel with the maximum *F*-value). The zero level corresponds to the estimated mean activation during rest periods. The circle indicates that the difference between seasons shows a trend (°*p* < 0.1) and the star indicates statistically significant difference between seasons (^*^p < 0.05).

To statistically confirm the seasonal effects observed visually in right NCM, we compared the differential BOLD response between SPEC and INDIV stimuli between the two seasons [(INDIV—PT) minus (SPEC—PT)]_breeding_ vs. [(INDIV—PT) minus (SPEC—PT)]_non−breeding_ using a repeated measures ANOVA (*N* = 5). A significant seasonal change in differential BOLD response between SPEC and INDIV stimuli was observed in a cluster located in the caudal part of the right NCM (*F*_max_ = 17.94, *p* = 0.022) (Figure 5A). This change was due to a significant seasonal change in activation by SPEC whistles (*t*_max_ = 2.42, *p* = 0.015), and a trend for a seasonal change in activation by SPEC warbling (*t*_max_ = 1.62, *p* = 0.075). Activation by INDIV songs (INDIV whistles: *t*_max_ = 0.80, *p* = 0.236; INDIV warbling: *t*_max_ = 1.37, *p* = 0.113) and activation by PT (PT_INDIVsession_: *t*_max_ = 0.68, *p* = 0.268; PT_SPECsession_: *t*_max_ = 1.34, *p* = 0.118) remained unchanged across seasons (Figure 5B).

### Lateralization

**Figure 6:**
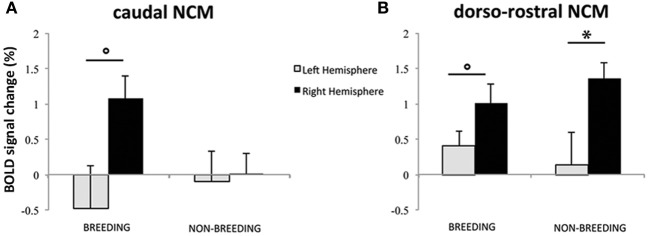
**Lateralization of differential song processing. (A)** Estimates of the relative amplitude of differential song responses in left and right caudal NCM. **(B)** Estimates of the relative amplitude of differential song responses in left and right dorso-rostral NCM. Positive values indicate that the region was more activated by INDIV than by SPEC. Negative values indicate that the region was more activated by SPEC than by INDIV. The error bars correspond to SEMs across subjects. Circles indicate that the difference between left and right NCM clusters shows a trend (*p* < 0.1); stars indicate that the difference between left and right NCM clusters is significant (*p* < 0.05).

In the analyses presented above, the differential BOLD response between SPEC and INDIV stimuli during the breeding season was observed in right caudal NCM and right dorso-rostral NCM but was not present on the left side (INDIV vs. SPEC in left caudal NCM: *p* = 0.646; in left dorso-rostral NCM: *p* = 0.120). During the non-breeding season, it was observed in right dorso-rostral NCM only and was not present on the left side (INDIV vs. SPEC on the left side: *p* = 0.234). To test for lateralization of these differential activations, we directly compared the left and right NCM clusters [defined as the functional regions identified by previous comparisons (Figures 3, 4) and their mirrored counterparts]. In caudal NCM, the differential activations elicited by INDIV vs. SPEC songs [(INDIV—PT) minus (SPEC—PT)] showed a trend toward right lateralization during (left vs. right: *t* = 2.032, *p* = 0.082, *N* = 8), but not outside (left vs. right: *t* = 0.261, *p* = 0.800, *N* = 8) the breeding season (Figure 6). In dorso-rostral NCM, the differential activations elicited by INDIV vs. SPEC songs showed a trend toward right lateralization during the breeding season (left vs. right: *t* = 1.977, *p* = 0.089, *N* = 8) and was significantly lateralized to the right during the non-breeding season (left vs. right: *t* = 2.398, *p* = 0.043, *N* = 8) (Figure 6).

## Discussion

Our results provide evidence that the auditory area NCM is clearly involved in conspecific song processing/categorization and that, in addition to morphological changes (De Groof et al., 2009), season also has a significant effect on differential song processing in a caudal sub-region of this area. We also observed that the differentiation between songs conveying species-specific and group information and songs conveying individual information seems to be biased toward the right hemisphere.

Evidence for the role of NCM in processing conspecific songs comes from electrophysiological studies (Chew et al., 1996) and studies of stimulus-driven expression of the immediate-early-gene ZENK, a marker of neuronal activity (Mello et al., 2004). In addition to being selective for conspecific songs, NCM neurons exhibit experience-dependent plasticity, and they are required for the formation of auditory memories (Mello et al., 2004; Terpstra et al., 2004; Bolhuis and Gahr, 2006; Phan et al., 2006; London and Clayton, 2008; Pinaud and Terleph, 2008). Moreover, in starlings, failure to correctly use songs whose elementary acoustic structure is otherwise species-typical leads to undifferentiated responses to these songs in NCM (George et al., 2010). Indeed, starlings that could hear but not interact with adults during early life are unable to differentiate individual whistles and warbling motifs not only in their vocalizations but also in their neural (NCM) responses to these songs, independently of the structural differences between these two types of songs. Although they do produce individual whistles and warbling motifs whose acoustic morphology is species-typical, these songs are not produced in species-typical sequences and NCM responses to these songs do not differ like they normally differ in wild-caught starlings (George et al., 2008). Using fMRI, we here confirm that, in wild-caught, adult male starlings, NCM shows differential responses to the functional classes of starling song (George et al., 2008), with songs bearing individual information being the most effective stimuli. Interestingly, we here differentiated 2 separate (sub)-regions of NCM that both display differential song processing during the breeding season: a dorso-rostral and a caudal region. Moreover, seasonal changes in differential song processing were observed only in the caudal part of NCM.

NCM as an anatomical region is quite large, and some researchers have treated it as one entity, presenting results from seemingly random parts of it (e.g., Duffy et al., 1999; Bolhuis et al., 2000, 2001). Efforts have been made to try and divide it into different anatomical or functional sub-regions, albeit not with a general consensus on how to divide it (Maney and Pinaud, 2011). Gentner et al. (2001) introduced the practice of dividing it into a dorsal and ventral part, however based on connectivity, electrophysiological responses, and neurochemical markers NCM may be better divided into rostral and caudal domains (Pinaud et al., 2006). For instance it has been demonstrated that, in zebra finches, immunoreactivity for aromatase, an enzyme that converts testosterone into estradiol, is mostly concentrated along the caudal boundary of NCM (Pinaud et al., 2006). Also the caudomedial part of NCM is rich in estrogen receptors (Gahr et al., 1993; Bernard et al., 1999; Gahr, 2001) in songbirds. Our results argue for a distinction between NCM rostral and caudal domains as suggested by Pinaud et al. (2006) and they show that the rostral and caudal domains of NCM may be functionally distinct (Matragrano et al., 2011).

We only found differential responses in NCM, none of the other regions in our study showed differential responses between songs conveying species information and songs conveying individual information. NCM is a region specialized for conspecific song processing (Mello et al., 2004) and seeing as we only looked for differential responses within certain conspecific songs, it is not illogical that it is the only region able to differentiate between certain types of conspecific song. We would expect that maybe CMM is also capable of differentiating between songs conveying species or songs conveying individual information, however, we do not see this in our results. CMM has been shown to be selective for conspecific song and it appears that it can differentiate between directed and undirected song in zebra finches (Woolley and Doupe, 2008).

The song control nuclei like HVC, Area X and RA are known to respond to bird's own song (Margoliash, 1983; Margoliash and Fortune, 1992; Mooney, 2000; Alliende et al., 2012). We have seen this also in an fMRI experiment with starlings (unpublished study) and zebra finches (Poirier et al., 2009). Since we did not have any bird's own song stimuli in this study it is likely that we did not see (differential) activations in these regions because of this.

The seasonal change in differentiated response to songs bearing species-specific and songs bearing individual information observed in caudal NCM was due to a seasonal change in neural responses to songs bearing species-specific information. Outside the breeding season, the BOLD response in caudal NCM was similar for all types of songs but, during the breeding season, BOLD response to songs bearing species-specific and group information was significantly different from that to songs bearing individual information. This change in neural processing shows interesting parallels with the change in social behavior and organization that can be observed in starlings across seasons. As we said earlier, whereas starlings spend most of the non-breeding season in flocks and roosts made of hundreds or thousands of individuals, they spend most of the breeding season in pairs or small groups (Verheyen, 1970; Hausberger, 1997). As social organization varies from bigger to smaller groups of starlings along the year, the relative importance of close and remote social interactions may vary accordingly. Thus, whereas individual vocalizations involved in close social interactions are likely to be important all over the year and in all kinds of contexts, species-specific and group vocalizations involved in remote social interactions may be more important in large groups and hence during the non-breeding season. Interestingly, whereas species-specific whistles are thought to play a role in inter-individual spacing in a breeding context [when males defend their nest boxes (Henry et al., 1994)], they may on the contrary play a role in inter-individual “attraction” and increase tolerance between birds that share the same (dialectal) variant within roosts, that is in a non-breeding context (Hausberger et al., 2008). This may explain that, while responses to songs bearing individual information remained constant across seasons, a decrease in the response to songs bearing species-specific and group information—leading to differentiated responses between the two—was observed during the breeding season.

It could be argued that the differential responses in NCM between songs bearing individual information and songs bearing species-specific and group information could be due to some overall spectral or temporal differences between these classes of stimuli. However, there are at least three reasons why this is very unlikely: (1) pure tones—whose spectral structure was more similar to that of species-specific whistles than to that of other stimuli—evoked the same kind of activation across seasons, (2) the responses to species-specific warbling showed seasonal differences that were similar to those observed for species-specific whistles although their spectro-temporal structure was very different from that of whistles, and (3) responses to individual whistles did not vary seasonally although their acoustic structure was more similar to that of species-specific whistles than to that of other stimuli. It is therefore very likely that (the seasonal changes in) NCM differential responses relied more on the stimuli's behavioral relevance or meaning (and their seasonal change) than on their acoustic structure. Moreover, season affects auditory processing in songbirds not only at the telencephalic level including NCM (Terleph et al., 2008; Phillmore et al., 2011) and the song control system (Del Negro and Edeline, 2001, 2002; Del Negro et al., 2005) but also at the peripheral level (Henry and Lucas, 2009; Caras et al., 2010). Auditory brainstem responses, which reflect activity generated by the auditory nerve and brainstem, seasonally change in a variety of songbirds (Henry and Lucas, 2009; Caras et al., 2010). One therefore has to take into account that changes that are observed in the central auditory system may be driven by changes in the periphery. However, since our data show the same BOLD response to pure tones across seasons, the seasonal effect we observed is unlikely to be merely due to changes at the periphery. Another possible, albeit unlikely, explanation of our findings could be that in NCM the activation pattern observed could be due to song novelty. All stimuli used were unfamiliar to the test subjects, but one could argue that species-specific songs are by nature less novel than individual songs. It has been shown that NCM is more active when birds are exposed to novel songs (Gentner et al., 2004; Woolley and Doupe, 2008), although these studies did not show a different response pattern within NCM for novel sounds, we showed here that it is altered seasonally only for caudal NCM, not for dorso-rostral NCM.

Regarding the stimuli used, ideally each subject would have been played a distinct exemplar of songs conveying species-specific and group information and songs conveying individual information in order to show that the distinct patterns of neuronal activity reflect responses to the proposed functionally distinct songs rather than to something unique to the limited set of recordings used (i.e., in order to avoid pseudoreplication). However, it should be noted that our results represent what is common between songs emitted by four different individuals. Indeed, the bold response mainly represents the neural activity elicited by what is in common between the two songs (AB) used for each stimulus class (the method, as implemented here, is not sensitive enough to detect something specific to one unique song). In addition, the main effect “songs bearing individual information vs. songs bearing species-specific and group information” within and between seasons was significant; the *post-hoc* tests showed that the difference was significant for whistles (songs from two individuals) and that there was a trend for warbling (songs from two other individuals), therefore representing an effect induced by songs coming from four different individuals.

What mechanism could be responsible for the change in functional neural responses over season? In seasonal songbird species, circulating gonadal hormones are modulated by photoperiod (Ball et al., 2004) and the baseline level of estradiol in NCM could thus fluctuate across seasons. This could in turn change the brain responses to auditory stimuli in this region. Caudal NCM has aromatase (the enzyme converting testosterone to estradiol) positive cells (Saldanha and Coomaralingam, 2005) and, within NCM, high concentrations of estrogen receptors (Gahr, 2001; Saldanha and Coomaralingam, 2005; Saldanha et al., 2011) and aromatase (Schlinger, 1997; Saldanha et al., 2000; Remage-Healey et al., 2010) have been found in songbirds species. Estrogens are known to affect GABAergic transmission in NCM (Tremere et al., 2009) and plasma steroids can significantly alter the catecholaminergic and serotonergic innervation of auditory regions in songbirds (Matragrano et al., 2011, 2012, 2013). This steroid-dependent innervation may be the mechanism by which sensory areas are tuned and their responses modulated in order to ensure a good match between social context of a signal and the subsequent behavioral response (reviewed by Maney and Pinaud, 2011). Recently, pharmacological manipulation of local inhibitory circuitry (GABAergic cells) in European starlings has shown that local inhibition plays an important role in NCM to enhance the encoding of behaviorally relevant songs (Thompson et al., 2013). This occurs by reducing response strengths and increasing selectivity concurrently, but independently of the sharpening of spectro-temporal receptive fields. This suggests that local inhibition does more than enhance neural tuning functions: it can modify neural coding to better represent behaviorally important stimuli. Such a mechanism may explain our results, especially as caudal NCM—where we observed seasonal changes in neural coding—is particularly rich in GABAergic cells (Pinaud et al., 2006).

The neural plasticity (at the neuron population level) we observed here across seasons could also be related to song learning. For starlings, which are open-ended learners, learning to recognize songs involves extensive neuronal plasticity (at the neuron level) in secondary auditory areas (Gentner and Margoliash, 2003; George et al., 2010; Thompson and Gentner, 2010). The non-differentiated BOLD response (between conspecific song stimuli) that we observed in caudal NCM during the non-breeding season may be compared to the high non-selective IEG response observed in juvenile male zebra finches during song learning. Zebra finches, who are closed-ended learners, only display a selective ZENK response for conspecific song when they are adult and their song is crystalized (Stripling et al., 2001). The development of this selectivity coincides with a rise in testosterone during song learning (Hutchison et al., 1984). In starlings, testosterone rises seasonally (Ball and Wingfield, 1987; Riters et al., 2002), as confirmed by our beak color data (see section Materials and Methods). Starlings could therefore be in a kind of “learning state” during the non-breeding season and in a kind of “crystallized state” during the breeding season. This is consistent with the higher variability of the song sequences (which may correspond to vocal exploration/practice) during the non-breeding season compared to the breeding season (when vocal performance may prevail) (Adret-Hausberger and Jenkins, 1988; Adret-Hausberger et al., 1990; Hausberger, 1991), which is also comparable to the higher variability in song of juvenile zebra finches compared to when they are adults (Miller et al., 2010).

Finally, our results suggest a greater involvement of the right hemisphere of starlings in differentiating songs conveying in individual information. This is in agreement with evidence for a dominant role of the right hemisphere in the recognition of individual conspecifics among birds and mammals (Vallortigara and Bisazza, 2002) and for right-hemisphere specialization for the recognition of faces in humans (Sergent and Signoret, 1992). It is also commonly accepted that language functions are processed asymmetrically in the human brain, with the left hemisphere dealing predominantly with verbal/semantic processing, and the right one with auditory individual recognition (von Kriegstein et al., 2003). Moreover, George et al. (2004) have provided the first clear evidence of hemispheric specialization in a songbird, by showing in the primary auditory area (Field L) of starlings a hemispheric specialization suggesting a greater involvement of the right hemisphere in the recognition of familiar conspecifics. The results of our study expand this to secondary auditory region NCM, however, it should be noted that we probably also observe right hemispheric specialization for Field L because the dorso-rostral NCM cluster likely also includes part of the sub-regions of Field L (i.e., L2b and/or L3) which do not have clear anatomical boundaries (except for L2a; dark region on our high resolution starling MRI image, see Figures 3, 4). In both studies, a predominant role of the right hemisphere processing signals bearing individual information was observed, supporting the idea that the right hemisphere plays a major role in individual recognition (Vallortigara and Bisazza, 2002). Asymmetries in song perception may shed light on the possible evolutionary origins of lateralization, especially in relation to language-like processes in the brain.

To conclude, we showed differential BOLD responses to behaviorally-defined classes of songs that convey different levels of social information in a secondary auditory area and, more importantly, we showed that, in male European starlings, season affects these responses significantly. Further research should examine the exact role that steroids may play in the seasonal change observed here. Seeing as seasons (and likely estrogens) may affect perception of social information, this could lead to a better understanding of human disorders characterized by social deficits [problems of interpreting communication signals like in autism (O'Connor, 2012)] that could be attributed to abnormalities of steroid receptors in auditory sensory areas of the brain (Sarachana et al., 2011). Songbirds may thus prove to be helpful models to develop treatments for individuals with these kinds of social deficits.

### Conflict of interest statement

The authors declare that the research was conducted in the absence of any commercial or financial relationships that could be construed as a potential conflict of interest.

## Abbreviations

3D: three-dimensional
ANOVA: analysis of variance
BOLD: blood oxygenation level dependent
CMM: caudomedial mesopallium
fMRI: functional magnetic resonance imaging
HVC: used as a proper name (previously higher vocal center)
IEG: immediate early gene
INDIV: individual songs (songs used in individual recognition)
LMAN: lateral magnocellular nucleus of anterior nidopallium
MLd: dorsal part of the lateral mesencephalic nucleus
NCM: caudomedial nidopallium
PT: pure tones
RA: nucleus robustus of the arcopallium
ROI: region of interest
SPEC: species-specific songs
ZENK: an acronym of immediate early genes
zif268: egr-1, NGFIA, and krox-24.
